# Socs1 and Socs3 degrades Traf6 via polyubiquitination in LPS-induced acute necrotizing pancreatitis

**DOI:** 10.1038/cddis.2015.342

**Published:** 2015-12-03

**Authors:** X Zhou, Z Liu, X Cheng, Y Zheng, F Zeng, Y He

**Affiliations:** 1 Department of Vascular and Thyroid, The Affiliated Hospital of Sichuan Medical University, Luzhou, Sichuan Province, P. R. China; 2 Department of Lymphoma and Myeloma, Division of Cancer Medicine, Center for Cancer Immunology Research, The University of Texas MD Anderson Cancer Center, Houston, TX, USA; 3 Department of Gastroenterology, The Affiliated Hospital of Sichuan Medical University, Luzhou, Sichuan Province, P. R. China; 4 Department of Biochemistry and Molecular Biology, Sichuan Medical University, Luzhou, Sichuan Province, P. R. China

## Abstract

Mechanisms involved in inflammatory development during acute pancreatitis (AP) are largely vague, especially in the transformation of acute edematous pancreatitis (AEP) into acute necrotizing pancreatitis (ANP). This current study aims to investigate the functions of Traf6 in different AP models *in vitro* and *in vivo*, and to identify the possible regulatory mechanism in the progression of inflammation from mild to severe. Our data revealed that the level of Traf6 expression was significantly increased in the mild AP induced by caerulein, and the upregulation of Traf6 played a protective role in acinar cells against caerulein-induced apoptosis. In contrast, only Traf6 protein but not mRNA was downregulated in the severe ANP induced by combination treatment of caerulein and LPS. Mechanistic studies showed that LPS upregulated the levels of Socs1 and Socs3 expressions in acinar cells, Socs1 and Socs3 interacted Traf6 directly and degraded Traf6 protein via polyubiquitination, thereby counteracted the protective function of Traf6. *In vivo* study further showed that combination treatment of caerulein and LPS failed to induce an ANP model in the TLR4 knockout mice, and the level of Traf6 expression in the pancreatic tissues remained the same as that from the acute edematous pancreatitis (AEP) mouse. Taken together, our study reveals that Traf6 functioned as a protective factor in the progression of AP, and LPS-induced Socs1 and Socs3 exacerbate mild AP to severe AP, which provides evidence for developing a new therapeutic target to combat AP.

Among the gastrointestinal diseases, acute pancreatitis (AP) accounts for the most emergency visits in the clinic, and the annual incidence is on the rise in the United States.^1^ This inflammatory disease has many severe complications, which also affects millions of patients around the world.^2^ One of the hallmarks of AP is parenchymal cell death: while the mild AP is generally associated with extensive apoptotic cell death, severe AP is primarily associated with necrosis and little apoptosis.^3^ Despite increasing researches in the latest decades, the mechanisms of cell death responsible for the pancreatic inflammation are not well elucidated, and the treatment remains ineffective. Therefore, it is important to uncover the regulatory mechanisms underlying the cell death during AP.

Toll-like receptors (TLRs) are a family of receptor proteins that can recognize structurally conserved motifs from microbes.^4^ Being the first line of host defense and essential for inflammatory initiation, TLRs have been suggested to be potentially related to the onset and progression of AP.^5, 6^ A cascade of downstream signal molecules is activated by TLRs, leading to the nuclear translocation of NF-κB and activator protein 1 (AP-1), and the release of inflammatory mediators. One of the key molecules in the TLR signaling pathway is the tumor necrosis factor receptor-associated factor 6 (Traf6). Mammalian Traf6 protein is highly conserved and consisted of an N-terminal Zn Ring finger domain, a series of five Zn-finger domains, a coiled-coil TRAF-N domain, and a C-terminal TRAF-C domain. As the only Traf family member which participates in signal transduction of the tumor necrosis factor (TNF) receptor superfamily and the interleukin-1 receptor (IL-1R)/TLR superfamily, Traf6 has been demonstrated to be involved in regulating cell death, survival, and cellular responses to stress.^7^ Deletion of Traf6 paralyzed NF-κB signaling and downregulated the productions of TLRs induced-cytokines.^8, 9^ Our previous study has shown that there is a correlation between the TLR4-Traf6 signaling and the severity of pancreatic inflammation in a mouse model with mild AP induced by caerulein. Thus, we speculate that Traf6 may function as an inflammatory adaptor to mediate apoptosis in pancreatic acinar cells.^10, 11^

A group of intracellular immune regulators, the suppressor of cytokine signaling (Socs) protein family, has been shown to negatively regulate TLR signaling via the modulation of signal transducer and activator of transcription (STAT) pathway.^12, 13^ There are eight members in the Socs family: Socs1 to Socs7 and cytokine-inducible Src homology 2 (SH2)-containing protein (Cis). Structurally, they all have a central SH2 domain and a C-terminal conserved domain termed the Socs box.^14^ The N-terminal domain varies in length, with shorter length in Socs2 and CIS, and longer length in Socs4. The N-terminal of Socs1 and Socs3 possesses a kinase-inhibitory region (KIR) domain that is critical for inhibition of kinase activity, and both proteins have E3 ubiquitin ligase activity.^15, 16^ Previous studies showed that Socs2 or Socs3 could target the Traf6 complex and inhibit signaling pathways associated with inflammation and innate immunity.^17, 18^ Both the expressions of Socs3 and Traf6 are reported to be increased in type 2 diabetes, which is a chronic low-grade inflammation, though the mechanism remains unclear.^19^ Furthermore, increased levels of Socs3 have been shown to inhibit inflammatory response by suppressing JAK2/STAT3 signaling in a caerulein-induced AP model both *in vitro* and *in vivo*.^20^ However, little is known about the interaction between Socs proteins and Traf6 in AP and the underlying mechanisms. Our current study aims to investigate the role of Traf6 in different types of AP *in vitro* and in the mouse experiments, and to identify the potential regulatory mechanism on how the inflammation could progress from mild to severe type.

## Results

### Traf6 protein increased in the mild AP but decreased in the severe AP

**Figure 1:**
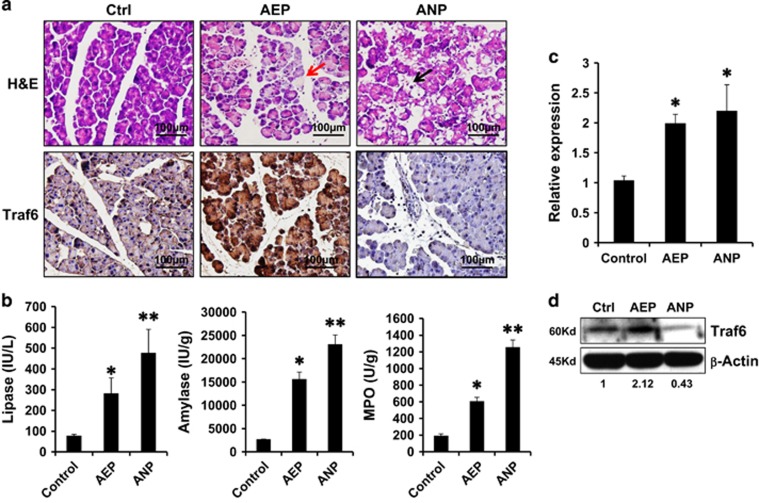
Traf6 levels in the acute edematous pancreatitis (AEP) and acute necrotizing pancreatitis (ANP) mice model. (**a**) Representative images of H&E stained pancreas tissue from control, AEP, and ANP mice (left panel), and the levels of Traf6 expression in the pancreatic tissues by immunohistochemistry staining (right panel). Red arrow, edematous acinar cells; black arrow, necrotizing acinar cells. (**b**) Serum MPO, amylase, and lipase levels in mice from Control, AEP, and ANP groups. (**c**) Detection of *Traf6* mRNA expression by real-time PCR or (**d**) Traf6 protein expression by western blotting analysis in pancreatic tissues from Control, AEP, and ANP groups. Actin served as a loading control. Values under the blots are ratios of Traf6/Actin and represent three independent assays. Representative results of three independent experiments are shown.*P<0.05 *versus* control. **P<0.01 *versus* control. Magnification, × 40. Bar size, 100 *μ*M

Mouse AP was induced by caerulein injection for the AEP model and additional LPS injection for the ANP model. Control mice (Ctrl) received PBS injection. The specimens were collected immediately after euthanization. Hematoxylin and eosin (H&E) staining showed the characteristic pathological changes in AP, including interstitial edema, acinar vacuolization, inflammatory cell infiltration, and pancreatic necrosis in both the AEP mice and the ANP mice (Figure 1a, red and black arrows). The severity of AP can be evaluated by the levels of serum amylase and lipase, and both were significantly increased in the two AP groups as compared with those in the control mouse. In addition, myeloperoxidase (MPO) is released by active neutrophils in the inflamed tissue, makes pancreatic MPO activity a good indicator for inflammation infiltration. As shown in Figure 1b, serum amylase, lipase, and MPO activities in the ANP group were significantly higher than those in the AEP group and in the control group. Our previous study has shown that Traf6 is involved in the inflammatory process occurred in AP; therefore, we examined the levels of Traf6 in the mild and severe AP model. In line with our previous results,^10^ the expressions of Traf6 were detected both at the mRNA level and protein level. Real-time polymerase chain reaction (PCR) data showed increased levels of Traf6 in both the ANP group and the AEP group (Figure 1c). However, western blotting showed that the Traf6 protein level was decreased in the AEP group compared with that in the control group and in the ANP group (Figure 1d), and this is consistent with the immunohistochemical staining using antibody against Traf6 (Figure 1a, right panel).

### Traf6 played a protective role in acinar cells from caerulein-induced apoptosis

**Figure 2:**
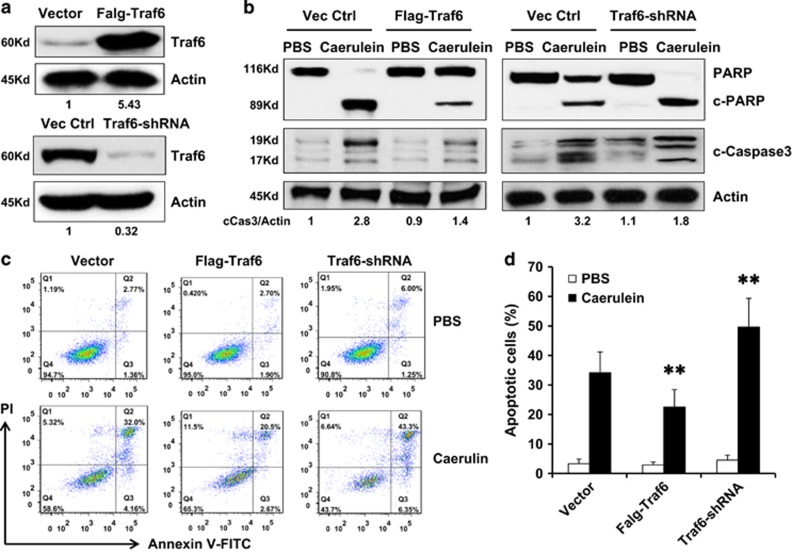
Protective effect of Traf6 in the acinar cell from caerulein-induced apoptosis. (**a**) Western blotting analysis showed the levels of Traf6 protein in the mouse acinar cell line 266-6 with stable expression of pcDNA-flag-Traf6 (Flag-Traf6) to overexpress Traf6 or shRNA targeting Traf6 (shRNA-Traf6) to knock down Traf6. Actin served as a loading control. Using those 266-6 cells with Flag-Traf6 or shRNA-Traf6 was treated with 2 *μ*M caerulein for 24 h, and cells were subjected to western blotting analysis and flow cytometry analysis. Cells with pcDNA-flag or with non-target shRNA served as respective control. (**b**) Western blotting analysis showed the levels of cleaved-PARP and cleaved-caspas-3. Actin served as a loading control. (**c**) Flow cytometry analysis using Annexin V-FITC and propidium iodide (PI) staining to detect apoptotic 266-6 acinar cells with Flag-Traf6 or shRNA-Traf6, and the summarized data are shown in (**d**). Representative results of at least three experiments are shown. **P<0.01

Our *in vivo* data showed that Traf6 expression was elevated in the AP process, we wondered if Traf6 behaves similarly *in vitro* in caerulein-induced AP. Mice pancreatic acinar cells 266-6 were transfected with pcDNA-flag-Traf6 (Flag-Traf6) plasmid or shRNA targeted Traf6 (Traf6-shRNA) in order to overexpress or knockdown the expression of Traf6, with pcDNA-flag (Vector) or non-target control shRNA (NT-control) as respective control. The stable cell lines were screened and established. As shown in Figure 2a, the level of Traf6 expression was dramatically increased in cells with Flag-Traf6 compared with that of in control cells, and its level was greatly reduced in cells with Traf6-shRNA compared with that of in NT-control cells. Cells were first treated with PBS or 2 *μ*M of caerulein for 48 h and then their abilities to induce apoptosis were analyzed. Western blotting showed that caerulein treatment induced PARP and caspase-3 cleavage in the control cells, and the presence of overexpressed Traf6 in Flag-Traf6 cells significantly decreased the level of cleavages (Figure 2b, left panel). Moreover, cleavage of those two apoptotic markers in the Traf6-shRNA cells was much higher than that in the NT-control cells (Figure 2b, right panel). Those cells were also subjected to Annexin V assay to assess apoptosis. The percentage of the apoptotic cells was much lower in the Flag-Traf6 cells, and much higher in the Traf6-shRNA cells than that in their respective control cells (Figures 2c and d). As previously reported, Traf6 exerts protective function largely by regulating downstream genes which plays roles in antiapoptosis, such as Bcl2 and Bax;^21^ therefore, we also validated the mRNA levels of Bcl2 and Bax in response to overexpression and knockdown of Traf6 gene, and confirmed that Bcl2 and Bax were downstream target of Traf6 (data not shown).

### LPS suppressed the elevated level of Traf6 induced by caerulein in 266-6 cells

**Figure 3:**
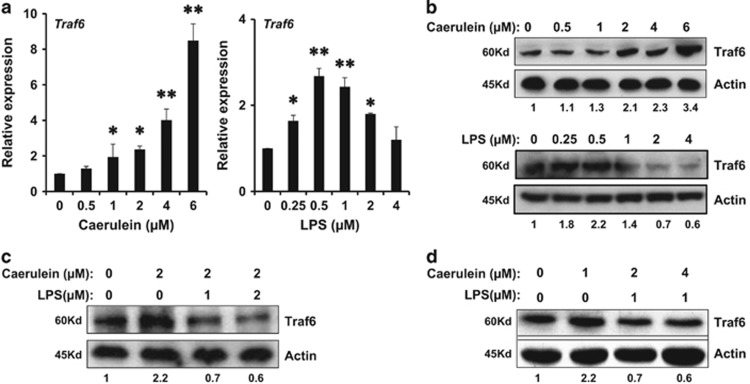
LPS attenuated the elevated Traf6 level in acinar cells induced by caerulein. (**a**) Quantitative PCR showed the relative expression levels of Traf6 in the mouse acinar cell line 266-6 in the presence of various concentrations of caerulein or LPS for 48 h. (**b**) Western blotting analysis showed the levels of Traf6 in 266-6 cells after a 48-hour treatment of different concentrations of caerulein (upper panel) or different dosages of LPS (lower panel). (**c**) Western blotting analysis showed the levels of Traf6 in 266-6 cells treated with 2 *μ*M of caerulein and increasing doses of LPS, or (**d**) treated with 1 *μ*M of LPS and increasing doses of caerulein for 48 h. Values under the blots are ratios of Traf6/Actin and represent three independent assays. Representative results of at least three experiments are shown. *P<0.05; **P<0.01

Since the expression level of Traf6 was decreased in the ANP model with addition of LPS *in vivo*, we next investigated the effect of LPS on Traf6 expression *in vitro*. 266-6 cells were treated with various concentrations of caerulein (0.5–6 *μ*M) or LPS (0.25–4 *μ*M), and the Traf6 expression was elevated in a dose-dependent manner as verified by real-time PCR and western blotting analysis (Figure 3a, left panel and b, upper panel). However, when the 266-6 cells were treated with LPS (0.25–4 *μ*M), the levels of Traf6 expression were elevated at lower dosages (≤0.5 *μ*M), but dropped significantly when the concentration of LPS was greater than 1 *μ*M (Figure 3a, right panel and b, lower panel). To examine whether LPS played a direct role in the inhibition of Traf6 expression, cells were treated with caerulein or LPS alone, or both in different combinations for 48 h, and collected for western blotting analysis. We found that while treatment of 2 *μ*M caerulein alone increased the levels of Traf6 expression significantly, the combination of 2 *μ*M caerulein and 1 *μ*M LPS suppressed the activation of Traf6 by caerulein (Figure 3c). Moreover, in the presence of 1 *μ*M LPS, increased concentrations of caerulein failed to activate the Traf6 level in the 266-6 cells (Figure 3d), suggesting that LPS could negatively regulate the levels of Traf6 expression in caerulein-induced AP *in vitro*.

### LPS induced the binding of Socs1 and Socs3 to Traf6 and the degradation of Traf6 protein via hyperubiquitination

**Figure 4:**
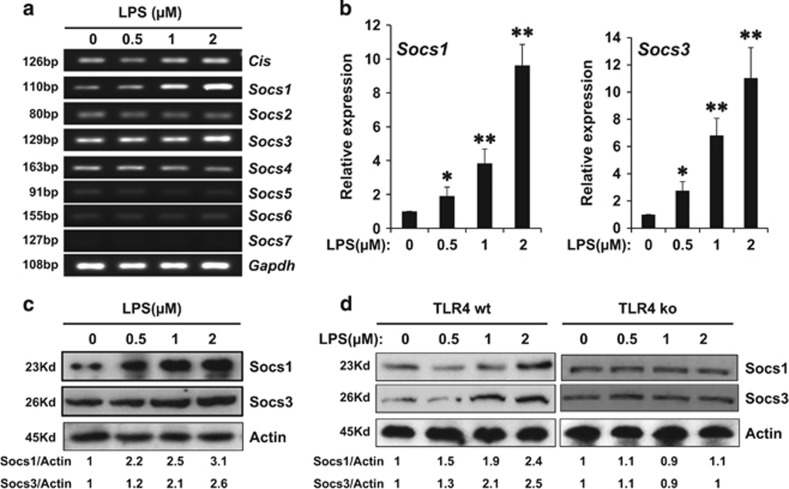
LPS treatment induced the Socs1 and Socs3 expressions in mouse acinar cells. (**a**) Real-time PCR was used to screen the mRNA levels of the eight Socs family members in 266-6 cells after a 48-h treatment with different dosages of LPS. Quantitative PCR (**b**) and western blotting analysis (**c**) confirmed the levels of Socs1 and Socs3 in 266-6 cells treated with various concentrations of LPS. Acinar cells isolated from TLR4 wt and ko mice were treated with increasing concentrations of LPS, and levels of Socs1 and Socs3 were evaluated by western blotting analysis (**d**). Actin served as a loading control. Values under the blots are ratios of Socs1 (upper) or Socs3 (lower) to Actin and represent three independent assays. Representative results of at least three experiments are shown. *P<0.05; **P<0.01

Previous study showed that the Socs family of proteins played feedback roles in inflammatory signal transduction.^22^ Thus we explored whether Socs proteins are critical in the suppression of Traf6 protein in LPS-mediated acute necrosis pancreatitis. The expressions of all eight members of Socs family of genes were examined in LPS-treated 266-6 cells using RT-PCR. We found that CIS, Socs1, Socs3 and Socs4 were expressed in the 266-6 cells, and the rest of four showed only decimal expressions. When LPS was added, the expressions of Socs1 and Socs3 increased significantly, especially at a concentration of 2 *μ*M, while the rest remained mostly unchanged (Figure 4a), suggested Socs1 and Socs3 may be involved in the LPS-mediated modulation of ANP. This is confirmed by examining the expression levels of Socs1 and Socs3 using real-time PCR and western blotting analysis, which both showed dose-dependent increases after LPS treatment (Figures 4b and c). Since LPS binds the receptor TLR4 and triggers innate immune responses in inflammation process, we are wondering if addition of LPS would affect the expression of Socs proteins *in vivo* with or without TLR4 expression. Using TLR4 wild type (TLR4 wt) and the knockout (TLR4 ko) mice, the primary acinar cells were isolated from pancreas, and various concentrations of LPS treatment were given for 48 h. Western blotting analysis showed that the expressions of Socs1 and Socs3 were upregulated by LPS treatment in a dose-dependent way in the TLR4 wt mice, in line with the results obtained from 266-6 cells. However, the protein levels of Socs1 and Socs3 were not elevated in LPS-treated acinar cells isolated from TLR4-KO mice (Figure 4d), confirming that Socs proteins are a downstream target for LPS modulation of ANP.

**Figure 5:**
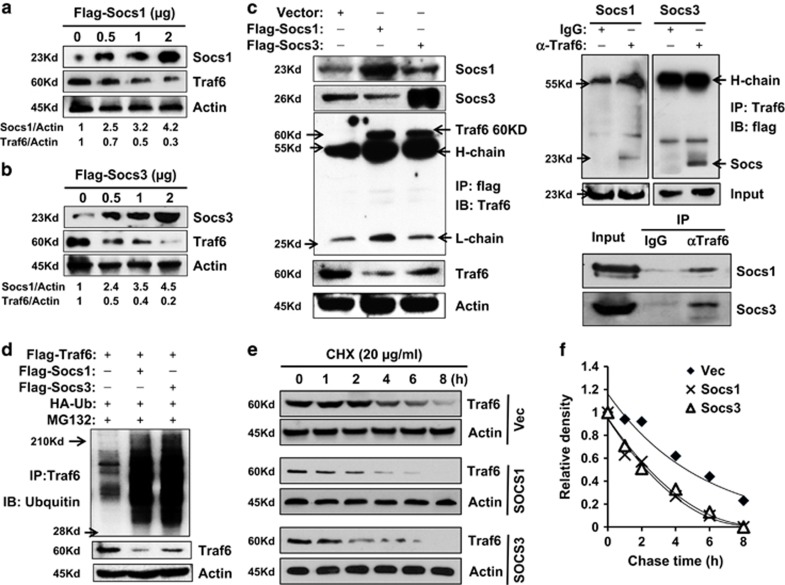
Socs1 and Socs3 interact with and hyperubiquitinate Traf6 protein. Various amounts of pcNDA-flag-Socs1 (Flag-Socs1) or pcDNA-flag-Socs3 (Flag-Socs3) were transiently transfected into 266-6 cells for 48 h, and cell lysates were collected for analysis: (**a**) western blotting analysis showed the expression of Socs1 and Traf6; (**b**) western blotting analysis showed the expression of Socs3 and Traf6. (**c**) Immunoprecipitation assay showed the interaction between Socs1 with Traf6 or Socs3 with Traf6, respectively. Left, exogenous flag-Socs1 and flag-Socs3 were expressed in 266-6 cells and antibody against flag was used to pull down the complex, and antibody against Traf6 was used for the detection; upper right, exogeneous Traf6 was expressed in 266-6 cells and antibody against Traf6 was used to pulldown and Socs1 and Socs3 antibodies were used for immunoblotting; lower right, interaction between endogenous Traf6 and Socs1/Socs3 in the primary acinar cells isolated from wild-type mice; 10% total protein was loaded as input control. (**d**) Immunoprecipitation assay showed Socs1 and Socs3 accelerated the Traf6 degradation via Traf6 hyperubiquitination. An antibody against Traf6 was used to pull down the complex, and antibody against ubiquitin was used for the detection of Traf6 hyperubiquitination. (**e**) Exogenous Traf6 was expressed in 266-6 cell alone or combined with flag-Socs1 or flag-Socs3 for 48 h, then 20 *μ*M of CHX was admitted for up to 8 h, and half-life of Traf6 was analyzed. (**f**) Half-life of Traf6 protein was analyzed using densitometry assay. Representative results of at least three experiments are shown

Next, we further our investigation onto the relationship between Socs and Traf6 proteins in the acute pancreatic inflammation process. The 266-6 cells were transfected with pcDNA-flag-Socs1 (Flag-Socs1) or pcDNA-flag-Socs3 (Flag-Socs3) plasmids to overexpress Socs1 and Socs3 protein, respectively. After 48 h, the levels of Traf6 expression were examined using western blotting analysis. As shown in Figures 5a and b, the increased levels of Socs1 and Socs3 expression corresponded to the downregulation of the levels of Traf6 expression. To investigate whether Socs protein could directly interact with Traf6 protein, the immunoprecipitation assay was performed. The 266-6 cells were transfected with pcDNA-flag (Vector), pcDNA-flag-Socs1 or pcDNA-flag-Socs3 plasmids for 48 h, lysed with RIPA buffer, and pulled down with an antibody against flag tag. The antibody against Traf6 was used in the immunoblotting to detect the protein–protein interactions. In Figure 5c, western blotting from the top two panels confirmed the overexpression of Socs proteins in respective cells. More importantly, the immunoprecipitation assay showed that both the Socs1 and Socs3 protein interacted with Traf6, and the levels of Traf6 expression were decreased when the levels of Socs1 or Socs3 were increased as compared with that of in cells with only the Vector (Figure 5c, left panel). On the other hand, when the exogenous Traf6 was overexpressed in the 266-6 cells then pull down by IP antibody, interaction with Socs1 and Socs3 could be validated via immunobloting (Figure 5c, upper right); meanwhile, association of Traf6 with Socs1 and Socs3 in the primary acinar cells was also examined in our study, as shown in Figure 5c (lower right), binding of endogenous Traf6 to Scos1 and Socs3 could be verified in an immunoprecipitation assay. Next, the 266-6 cells were co-transfected with plasmids containing Traf6, HA-ubiquitin, Socs1 and/or Socs3 for 48 h, 10 *μ*M MG132 was added for additional 4 h, and the ubiquitination status of Traf6 protein was analyzed. As shown in Figure 5d, increased levels of Socs1 or Socs3 significantly upregulated the ubiquitination level of Traf6 protein, and the levels of total Traf6 protein were decreased accordingly when compared with that in the control. Furthermore, since Socs1 and Socs3 affect Traf6 protein stability, we employed experiments to test the half-life of Traf6 in response to Socs1 and Socs3 overexpression. 266-6 cells transfected with Traf6 expressing plasmid and empty vector, or Socs1 and Socs3 expressing vectors for 48 h, were treated with 20 *μ*M of protein biosynthesis inhibitor cycloheximide (CHX) for different time (0–8 h), and degradation of Traf6 protein was chased. Our results confirmed that the half-life of Traf6 protein was about 6 h,^23^ but with Socs1 or Socs3, the half-life of Traf6 decreased dramatically to about 3 h (Figures 5e and f). These results suggested that Socs1 and Socs3 bind directly to Traf6 protein, and accelerate the Traf6 degradation via hyperubiquitination.

### Acute necrosis pancreatitis could not be induced and the Traf6 protein could not be regulated by Socs1 and Socs3 in the TLR4 knockout mice

**Figure 6:**
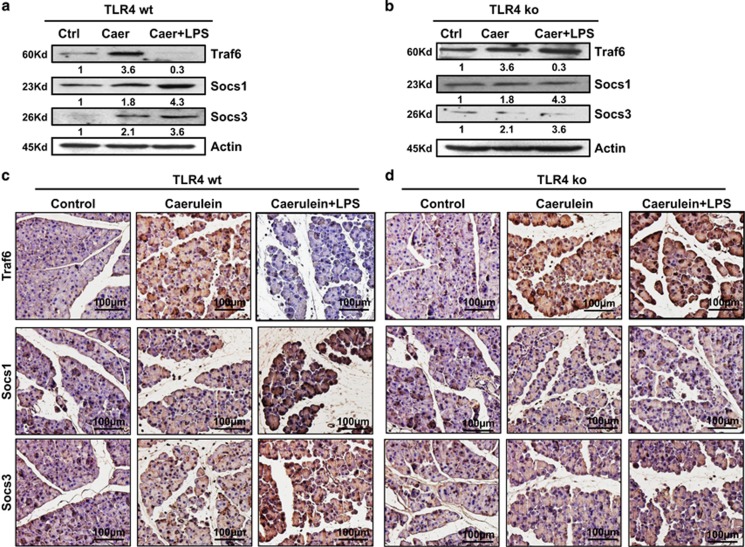
Expression of Traf6 and Socs protein in the TLR4 wt and TLR ko mice. TLR4 wt and TLR4 ko mice were treated with 5 *μ*g/kg caerulein alone or with combination of 5 *μ*g/kg caerulein and 1 *μ*M LPS. Western blotting analysis showed the protein levels of Traf6, Socs1 and Socs3 in the TLR4 wt (**a**) and TLR4 ko (**b**) mice with induction of AEP and ANP. Immunohistochemistry staining for protein levels of Traf6, Socs1, and Socs3 in TLR4 wt (**c**) and TLR4 ko mice (**d**) Magnification, × 40. Bar size, 100 *μ*M

Subsequently, caerulein treatment alone or in combination with LPS treatment was given to the TLR4 wt and TLR4 ko mice to induce AEP or ANP. The pathological changes were confirmed by histochemical staining, MPO assay and enzymology assay. In the TLR4 wt mice, both AEP and ANP mice models were successfully induced, while only the AEP mice model could be induced in the TLR4 ko mice (data not shown). The levels of Traf6 protein in the acinar cells from the TLR4 wt mice were significantly downregulated with combination treatment of caerulein and LPS as compared with those with caerulein treatment only, while the levels of Traf6 protein in the acinar cells were similar among TLR4 ko mice with AEP and ANP (Figures 6a and b). As predicted, the levels of Socs1 and Socs3 protein were elevated only in the acinar cells from TLR4 wt mice but not from TLR4 ko mice with combination treatment of caerulein and LPS (Figures 6a and b). The specimens of pancreatic tissues from TLR4 wt (Figure 6c) and TLR4 ko mice (Figure 6d) were also collected, and immune stained with antibodies against Traf6, Socs1, and Socs3. Their expression levels detected by immunostaining were in line with the results from RT-PCR and western blotting, indicating that TLR4 played an important role in LPS-induced ANP through mediating the levels of Traf6 expression.

## Discussion

Traf6 is the only Traf family member that participates in signal transduction of TNF receptor superfamily and the IL-1R/TLR superfamily,^24^ and has been demonstrated to be involved in regulating cell death, cell survival, and inflammation.^25, 26^ The involvements of Traf6 in acute or chronic inflammatory conditions is specially intriguing, with a wide range from acute lung injury,^27, 28^ to cerebral ischemia/reperfusion,^29^ chronic vascular inflammatory disease,^30^ *Helicobacter pylori*-related chronic gastritis,^31^ etc. Our previous study also demonstrated that Traf6 plays a protective role in caerulein-induced mild AP *in vivo*.^10^ However, the detailed function of Traf6 and its underlying mechanism still remains unclear, especially its effect on the progression of mild AP to severe AP.

In this study, we first evaluated the expression of Traf6 in the pancreatic tissues extracted from mice with the AEP and the acute necrosis pancreatitis. Our RT-PCR results showed that the expression of Traf6 was activated in both the AEP and the ANP mice, and there was no significant difference between them. However, results from western blotting analysis clearly showed a decrease in the expression of Traf6 protein in the ANP mice than that in the AEP mice, suggesting that Traf6 is associated with the inflammatory procession of mild AP to severe AP, and the levels of Traf6 was modulated not at the transcriptional level but at the post-translational level. Further functional study of Traf6 in mouse acinar 266-6 cells confirmed its protective role in the caerulein-induced apoptosis. A series of articles have indicated that Traf6 regulates apoptosis-associated signaling pathways: He *et al.*^32^ reported the human Traf6 could directly mediate programmed cell death in a caspase-8 dependent pathway; another study revealed that inhibition of Traf6 expression reduced activation of caspase-3 and inhibited inflammatory response in LPS-treated rat renal tubular cells;^33^ Zhang *et al.*^34^ also demonstrated that Traf6 participated in LPS-induced cardiac cell injury and apoptosis via Akt2 ubiquitination; Traf6 have also been shown to protect immature and mature DC apoptosis during the differentiation, thus promotes DC cells survival.^26^ Our previous study has revealed similar protective function of Traf6 in pancreatic acinar cell apoptosis induced by caerulein treatment. In the current study, we revealed for the first time that Traf6 functioned as an adaptor in the progression of mild pancreatitis into severe pancreatitis through degradation of Traf6 protein. Our study also showed that while the caerulein treatment could induce the expression of Traf6, LPS worked in a different manner. In the presence of lower concentrations (under 0.5 *μ*M) of LPS, Traf6 can be recruited and activated through LPS binding to the TLR4 receptors via MyD88-dependent pathways.^35^ However, when LPS concentration is higher (>1 *μ*M), the Traf6 level was suppressed in acinar cells in a dose-dependent manner. This may explain why in the mice with acute necrosis pancreatitis, which was induced by the combination treatment of caerulein and LPS, the Traf6 level was lower compared with those in the AEP. Therefore, our research reveals a potential dual role for LPS in the regulation of Traf6 expression. Nevertheless, in our study, we found an interesting phenomenon that caerulein alone also induced the expression of Socs1 and Socs3 in the AEP model but did not degrade Traf6 protein. A possible hypothesis is that caerulein only stimulates Socs expression at a limited degree, and due to the complicated interaction with other signaling pathways the activity of Socs is suppressed, therefore the Traf6 protein will not be degraded; under LPS stimulation, Socs1 and Socs3 expressions increase dramatically and balance of other signaling pathways is broken, Socs activity and binding to Traf6 is invoked, therefore Traf6 protein will be degraded. However, mechanism underlying this pathogenesis is still unclear, the detailed mechanism needs to be further investigated.

Although inflammation is an essential mechanism for self-protection, but excessive inflammatory activation is harmful; therefore, strict negative feedback regulations come in as important modulators. As endogenous negative regulators, CIS/Socs family inhibits LPS-induced inflammatory cascades with different mechanisms.^36, 37^ For example, increased Socs3 could inhibit inflammatory response by suppressing the JAK2/STAT3 signaling pathway in the caerulein-induced AP model.^20^ In this study, we examined the expression patterns of all eight CIS/Socs family members, and found that the levels of Socs1 and Socs3 expression were increased most significantly after LPS treatment in mice acinar cells. More interestingly, we found that the elevation of Socs1 and Socs3 was only observed in primary acinar cells isolated from TLR4 wt mice but not in those from TLR4 ko mice, further confirmed that upregualtion of Socs1 and Socs3 were induced by LPS and mediated through TLR4. Recent study reveals that Socs proteins inhibit signaling pathways associated with inflammation and innate immunity by targeting the Traf6 complex via ubiquitination modification and proteasome-dependent degradation of Traf6.^38^ Another study also showed that Socs3 inhibits interleukin-1 signaling by targeting the Traf6/TAK1 complex in the type II diabetes.^17^ But whether Traf6 protein is a target for Socs proteins in the AP is unknown. Thus, we hypothesize that Socs could downregulate the levels of Traf6 expression in the AP and contribute to the impairment of the pathophysiological processes. Our results confirmed this hypothesis both *in vivo* and *in vitro*. In the mice acinar 266-6 cells, overexpressing either Socs1 or Socs3 protein suppressed the levels of Traf6 expression in a dose-dependent manner. In the TLR4 wt mice, the upregulation of Socs1 and Socs3 protein leads to the downregulation of the Traf6 expression with combination treatment of caerulein and LPS. However, the same treatment did not affected levels of Socs1 and Socs3 in the TLR4 ko mice. Therefore, the level of Traf6 expression is not downregulated neither. In addition, Socs1 and Socs3 are known to function as part of a multi-subunit E3 ubiquitin ligase complex, and mark target proteins for proteasomal degradation.^39, 40^ Using immunoprecipitation assays, we found that both Socs1 and Socs3 could integrate Traf6 to form a complex. Moreover, ubiquitination of Traf6 was enhanced in cells overexpressing Socs1 or Socs3, resulting in the accelerated degradation of Traf6.

In summary, our study strongly suggested that Traf6 exerts protective effect by inhibiting acinar cell apoptosis during AP, LPS-induced Socs1 and Socs3 lead to the degradation of Traf6 via hyperubiquitination, and exacerbate pancreatic inflammation from mild to severity. Since uncontrolled inflammatory responses are central to the deterioration of AP, causing enormous burdens and increased morbidity for patients, our results showed that Traf6 is a key modulator in pancreatitis progress and a promising biochemical target for the treatment of AP.

## Materials and Methods

### Establishment of *in vivo* AP model

C57BL/10ScNJ (TLR4-KO) and C57BL/10SnJ (wt) mice were originally purchased from Jackson Laboratory (Bar Harbor, ME, USA), maintained, and bred in house at the Experimental Animal Center of Sichuan Medical University (Luzhou, China). Twenty male mice per group, 4–6 weeks of age, 20–25 g body weight at the onset of the experiment, were housed at 23 °C with a 12 : 12 h light/dark cycle and were provided standard rodent chow and tap water. The animal experiments were approved by The Animal Care and Welfare Committee of Sichuan Medical University, and conducted according to the guidelines of the Local Animal Use and Care Committees of Luzhou as well as the National Animal Welfare Law of China.

The two AP mouse models were established as previously described.^41, 42^ Briefly, mild AP (AEP) was induced by 7 consecutive hourly i.p. injection of 50 *μ*g/kg caerulein (Sigma); severe AP (acute necrotizing pancreatitis, ANP) was induced by 13 consecutive injections of caerulein, followed by one dose of 15 mg/kg LPS. PBS injection served as control. Three hours after the last injection, mice were anesthetized with sodium pentobarbital and tissues were removed.

### Myeloperoxidase assay and enzymology examination

Myeloperoxidase (MPO) activity was measured using Chromatometric kit (Nanjing Jiancheng Bioengineering Institute, Nanjing, China) according to the manufacturer's protocol. Samples were collected as previously reported^43^ and MPO activity was determined by degrading 1 *μ*M of peroxide per minute at 25 °C. Results were expressed as units per gram weight (U/g) of wet tissue. Serum amylase and lipase levels were determined by using an automatic biochemical analyzer (Olympus AU5400, Tokyo, Japan) in the Biochemical Center of Affiliated Hospital of Sichuan Medical University and are expressed in terms of IU/l.

### Reagents, cell cultures, and plasmids

The antibodies against PARP, caspase-3, and *β*-Actin (all dilution 1 : 1000) were purchased from Cell Signaling Technology (Danvers, MA, USA); the antibodies against Traf6, Socs1, Socs3, and ubiquitin antibodies (all dilution 1 : 500) were purchased from Santa Cruz Biotechnology (Dallas, TX, USA); the mouse flag antibody (1 : 1000) was from Sigma Aldrich (St Louis, MO, USA). The MISSION shRNA-Traf6 plasmid was also purchased from Sigma Aldrich, and the plasmids expressing flag-Socs1, flag-Socs3, flag-Traf6, and HA-Ubiquitin were purchased from Addgene (Cambridge, MA, USA). The protein biosynthesis inhibitor cycloheximide (CHX), and the proteasome inhibitor MG132 were purchased from Sigma Aldrich.

Murine pancreatic acinar 266-6 cells were purchased from ATCC (Manassas, VA, USA), and cultured in Dulbecco's modified Eagle's medium supplemented with 10% fetal bovine serum, 100 U/ml penicillin, and 100 μg/ml streptomycin. For transient transfections into 266-2 cells, Lipofectamine 2000 (Life Technologies, Grand Island, NY, USA) was used according to the manufacturer's protocol, and lentiviral infection was used to knocking down the target genes as previously described.^43^ Isolation of dispersed primary pancreatic acinar cells were performed as previously described.^43^ Briefly, pancreatic tissues were dissected and gently grinded in a 70- *μ*M Nylon cell strainer (BD, Franklin Lakes, NJ, USA) and transferred to a conical tube. The suspension was shaken by hand in a 37 °C water bath for 5–10 min, washed and then the acinar cells were cultured in RPMI 1640 supplemented with 10% fetal bovine serum at 37 °C for further experiments.

### Real-time PCR

Total RNA was isolated from fresh tissue or cell using Trizol reagent (Life Technologies), and an aliquot of 1 *μ*g total RNA was reversely transcripted using a SuperScript II (Life Technologies) reverse transcriptase. qRT-PCR was performed using a StepOne Plus real-time PCR system (Applied Biosystems, Foster City, CA, USA) with SYBR Green Master Mix (Life Technologies). The primers used are listed below:

*Traf6* (132 bp): GTGGAGTTTGACCCACCTCT, TGCATCCCTTATGGATTTGA; *Socs1* (186 bp): GACACTCACTTCCGCACCT, GAAGAAGCAGTTCCGTTGG; *Socs2* (149 bp): GTCAGCTGGACCGACTAACC, TCCGTTTATCCTTGCACATC; *Socs3* (129 bp): GCAGGAGAGCGGATTCTACT, ACGCTCAACGTGAAGAAGTG; *Socs4* (163 bp): TTCCACACCCAGATCGACTA, AATCTTCCTGCGCTGAATCT; *Socs5* (188 bp): GTTCTGCAATTCCACAAACG, GATCTGAAGCAAATCTGGCA; *Socs6* (155 bp): ATGAACAGCCAGATGTGGAA, GAATCGTGACACTGGATTGG; *Socs7* (104 bp): GCTGCTCTATCCAGTGTCCA, GCAGATCCGGAATATGGTCT; *Cis* (129 bp): TGCATAGCCAAGACGTTCTC, GTGGGTGCTGTCTCGAACTA; *Actin* (120 bp): AGTGTGACGTTGACATCCGT, TGCTAGGAGCCAGAGCAGTA.

### Western blotting analysis

Western blot was carried out as previously described.^44^ Briefly, fresh tissue or cells were lysed in 1 × lysis buffer (Cell Signaling Technology). Fifty micrograms of total protein were separated via gel electrophoresis (Life Technologies, Carlsbad, CA, USA), and transferred onto a nitrocellulose membrane, immunoblotted with primary antibodies. *β*-Actin served as a loading control. All western blots were quantified by gray densitometry assay using Multi Gauge V3.0 software (Fuji Film, Tokyo, Japan). Values under each blot are shown as the ratios of target protein/Actin, and present the mean of three independent assays.

### Histological examination and immunohistochemistry

All specimens were paraffin-embedded, and the sections were deparaffinized first. H&E staining was used for routine histological examination. For immunohistochemical analysis, deparaffinized sections were stained with antibodies against Traf6, Socs1, or Socs3 at a dilution of 1 : 100, and counterstained with hematoxylin. All pictures were taken using a Nikon Eclipse E600 microscope (Nikon, Tokyo, Japan).

### Flow cytometry

Annexin V assay was performed as previously described.^45^ Briefly, 2 × 10^5^ murine pancreatic acinar 266-6 cells were trypsinized, washed, resuspended in 50 *μ*l of 1 × binding buffer containing 2 *μ*l fluorescein isothiocyanate (FITC)-Annexin V and 2 *μ*g/ml propidium iodide, and incubated for 15 min at room temperature. The samples were done in triplicate and analyzed using a flow cytometry from BD Biosciences (Franklin Lakes, NJ, USA).

### Immunoprecipitation experiments

Murine 266-6 cells were transfected with HA-ubiquitin (HA-Ub), Flag-Socs1, Flag-Socs3, and Flag-Traf6, alone or in combination. After 48 h, cells were lysed in RIPA buffer (0.5% deoxycholate and 1% Triton X-100, 50 mM Tris-HCl pH 8.0, 0.15  M NaCl, 2  mM EDTA, and 1 mM PMSF, 1 × protease inhibitor mixture) supplemented with protease inhibitor cocktail (Roche, Basel, Switzerland) and phosphatase inhibitors (10 mM NaF and 1 Mm Na_3_VO_4_). For primary acinar cells, after isolated and cultured for 24 h cells were subjected to experiments. Immunoprecipitations were performed using antibodies (2 *μ*g/mg total protein) against flag or Traf6 with protein A/G-agarose (50 *μ*l/mg total protein) (Santa Cruz, Dallas, TX, USA) at 4 °C, and immunoblotted with antibodies against Traf6, Socs1, Socs3 or ubiquitin (1 : 500), respectively.

### Statistical analysis

Experimental values are expressed as mean±standard error of the mean if not otherwise indicated. Statistical significance was analyzed using SPSS 10.0 software and determined by unpaired Student's *t*-tests and one-way analysis of variance. *P*-value <0.05 was considered statistically significant. All results were reproduced in at least three independent experiments.

## Abbreviations

Socs: suppressor of cytokine signaling
Traf6: tumor necrosis factor receptor-associated factor 6
AP: acute pancreatitis
AEP: acute edematous pancreatitis
ANP: acute necrotizing pancreatitis
